# What’s in a name? What we call growth hormone is much more than just a growth-related peptide

**DOI:** 10.20945/2359-3997000000183

**Published:** 2019-11-01

**Authors:** Cesar Luiz Boguszewski, Margaret Cristina da Silva Boguszewski

**Affiliations:** 1 Serviço de Endocrinologia e Metabologia Hospital de Clínicas Universidade Federal do Paraná Curitiba PR Brasil Departamento de Clínica Médica, Serviço de Endocrinologia e Metabologia do Hospital de Clínicas da Universidade Federal do Paraná (SEMPR), Curitiba, PR, Brasil; 2 Serviço de Endocrinologia e Metabologia Hospital de Clínicas Universidade Federal do Paraná Curitiba PR Brasil Departamento de Pediatria, Serviço de Endocrinologia e Metabologia do Hospital de Clínicas da Universidade Federal do Paraná (SEMPR), Curitiba, PR, Brasil

We welcome you to the fascinating world of one of the most intriguing peptides of our body: growth hormone (GH). GH is much more than a substance that promotes growth. It is a hormone that exhibits both anabolic and catabolic activities, has either diabetogenic or anti-insulin effects depending on the experimental condition, and has biological actions in virtually all human tissues and organs. The field of GH and GH-related disorders has undergone rapid advancements and innovations over recent years. Important contributions have been made by researchers in Brazil and worldwide, which are in part summarized in this special issue of *Archives of Endocrinology and Metabolism* . This edition comes in a perfect time to celebrate the Tenth International Congress of the Growth Hormone Research Society (GRS) and the Society for IGF Research, the biannual meeting of these international sister organizations devoted to the study of GH-IGF axis and related diseases, which will take place for the first time in Brazil, in November 2020.

In this special edition, Wasinski and cols. ( 1 ) present a review on the physiological GH action in the central nervous system, with particular focus in the potential role of GH in reproduction and energy balance based on very recent data coming from their own studies in animal models. They have demonstrated the importance of the brain as a target tissue for GH actions, with potential implications for metabolic homeostasis in health and disease. The biological effects of GH in specific tissues have aroused great interest and gained new insights in recent years from the studies in different tissue-specific GH receptor (GHR) knockout mice. List and cols. ( 2 ) have beautifully summarized the results of recent experiments performed by their group as well as by other researchers, addressing what is currently known about specific GH actions in liver, adipose tissue, muscle, immune and hematopoietic system, pancreatic β-cells, intestines, bone, and heart.

Chesnokova & Melmed ( 3 ) give us a detailed review about the involvement of GH and IGF-I in the different steps of tumor development, presenting a provocative hypothesis, based on their experiments with colon cells, on how excessive GH secreted by somatotroph pituitary adenoma cells, or locally induced-GH in non-tumorous tissue in response to inflammation, DNA damage or senescence, promotes “field cancerization” and creates a pro-tumorigenic environment.

Anabolic properties of GH have been widely associated with its capacity to enhance physical performance in sports. In his article, Ho ( 4 ) offers to us a comprehensive review of the literature, including his extensive expertise in this field, covering important physiological aspects of muscle structure and function, aerobic and anaerobic capacity, and their relationship with GH-IGF-I axis. He concludes from studies performed in fit people that GH improves anaerobic capacity but does not affect muscle strength or aerobic capacity, and as a consequence, GH is unlikely to benefit power or endurance sports and might be of benefit in sprint events.

GH has diabetogenic properties, increasing hepatic and renal glucose production and decreasing glucose uptake from peripheral tissues. It is also a lipolytic hormone. Accordingly, patients with GH deficiency (GHD) should exhibit hypoglycemia, but GHD is commonly associated with obesity and increased fat mass, which could oppose the hypoglycemic effect caused by reduced GH levels due to the development of insulin resistance. The balance of these two effects in relation to insulin sensitivity has generated discrepant results in the literature, which have been put into perspective by Garmes & Castillo ( 5 ), with an excellent review covering insulin signaling in the whole spectrum of GHD. van Bunderen and cols. ( 6 ) propose a personalized approach to GH replacement therapy in GHD adults, based on a review of the literature and their vast experience dating back to the early 1990s when first trials of GH therapy in GHD adults were published. They discuss the importance of individualized dose regimen, clinical and biochemical outcomes of GH therapy, and recent data on mortality of hypopituitary patients treated with GH. Adherence to GH therapy in both children and adults is likely a factor impacting responsiveness and outcomes. In an effort to improve patient adherence, long-acting GH formulations have been developed over the last 15 years or so, and Lal & Hoffman ( 7 ) update us with the most recent information and perspectives about these products, two already in the market in China and South Korea, and some others under clinical investigation in children and adults. These and other therapeutic advances are especially welcomed in the pediatric population, as new genetic defects in the GH-IGF-I axis causing growth retardation are revealed day after day, as well documented by Vasques and cols. ( 8 ) in their review, where the authors also discuss the upcoming challenges in the diagnostic and therapeutic management of short children.

In their chapter, Schilbach & Bidlingmaier ( 9 ) describe the evolution of analytical methods to measure biomarkers of GH action (mainly GH and IGF-I) and highlight the impact of methodological changes and biological variables on laboratory results. The most obvious implications of the variability in hormone measurements are in the establishment of cut-offs values for the diagnosis and follow-up of patients with GHD and acromegaly. They show that determination of serum IGF-I level is currently the most reliable and recommended parameter to use in clinical routine, while integrated, stimulated or suppressed GH measurements should be used as confirmatory value in GH-related disorders.

Acromegaly represents the best human model to understand the consequences of a continuous and prolonged exposure to high GH and IGF-I concentrations. Acromegaly might be associated with several comorbidities that contribute to an increased mortality in patients with active disease. Nevertheless, there have been great advances in the treatment of acromegaly and its comorbidities, with an important reduction and even normalization of mortality rates. This changing face of acromegaly is superbly presented by Kasuki and cols. ( 10 ), who summarize the current knowledge on the determinants of comorbidities and mortality in this intriguing disease. Since acromegaly patients are living longer, Jallad & Bronstein ( 11 ) have highlighted in their review the most striking differences in the clinical presentation and in the management of acromegaly in elderly individuals in relation to other age groups. In the last two or three decades, one remarkable advance in the acromegaly treatment has been the combination of drugs with different mechanism of action, which has opened a new perspective in the management of the disease. Coopmans and cols. ( 12 ) share with us the current position of combination medical therapy in acromegaly, based on the results of recent clinical studies and their own experience at the Erasmus Medical Center in Rotterdam. They discuss the potential additive and synergistic effects of the drugs and present what is currently known about efficacy and safety of this therapeutic modality.

We are truly indebted to all the authors for their invaluable contributions, providing us with outstanding and updated reviews that we appreciate very much to read (and certainly our readers will also do)! We thank them for their commitment with our journal. Last, but not least, we would like to thank the Editor-in-Chief for his support and Roselaine Monteiro da Silva and Sandra Regina Santana, from the Editorial Office, for their assistance in preparing this special issue.
